# Minding your steps: a cross-sectional pilot study using foot-worn inertial sensors and dual-task gait analysis to assess the cognitive status of older adults with mobility limitations

**DOI:** 10.1186/s12877-023-04042-6

**Published:** 2023-05-26

**Authors:** Vânia Guimarães, Inês Sousa, Eling D. de Bruin, Joana Pais, Miguel Velhote Correia

**Affiliations:** 1 Fraunhofer Portugal AICOS, Porto, Portugal; 2 Faculty of Engineering, University of Porto, Porto, Portugal; 3 Institute of Human Movement Sciences and Sport, Department of Health Sciences and Technology, ETH Zurich, Zurich, Switzerland; 4 Division of Physiotherapy, Department of Neurobiology, Care Sciences and Society, Karolinska Institute, Stockholm, Sweden; 5 OST - Eastern Swiss University of Applied Sciences, Department of Health, St. Gallen, Switzerland; 6 Neuroinova, Lda., Vila Nova de Gaia, Portugal; 7 EPIUnit - Institute of Public Health, University of Porto, Porto, Portugal; 8 Laboratory for Integrative and Translational Research in Population Health (ITR), University of Porto, Porto, Portugal; 9 INESC TEC (Institute for Systems and Computer Engineering, Technology and Science), Porto, Portugal

**Keywords:** Inertial sensors, Gait analysis, Cognitive impairment, Dual-task, Older adults, Mobility

## Abstract

**Background:**

Cognitive impairment is a critical aspect of our aging society. Yet, it receives inadequate intervention due to delayed or missed detection. Dual-task gait analysis is currently considered a solution to improve the early detection of cognitive impairment in clinical settings. Recently, our group proposed a new approach for the gait analysis resorting to inertial sensors placed on the shoes. This pilot study aimed to investigate the potential of this system to capture and differentiate gait performance in the presence of cognitive impairment based on single- and dual-task gait assessments.

**Methods:**

We analyzed demographic and medical data, cognitive tests scores, physical tests scores, and gait metrics acquired from 29 older adults with mobility limitations. Gait metrics were extracted using the newly developed gait analysis approach and recorded in single- and dual-task conditions. Participants were stratified into two groups based on their Montreal Cognitive Assessment (MoCA) global cognitive scores. Statistical analysis was performed to assess differences between groups, discrimination ability, and association of gait metrics with cognitive performance.

**Results:**

The addition of the cognitive task influenced gait performance of both groups, but the effect was higher in the group with cognitive impairment. Multiple dual-task costs, dual-task variability, and dual-task asymmetry metrics presented significant differences between groups. Also, several of these metrics provided acceptable discrimination ability and had a significant association with MoCA scores. The dual-task effect on gait speed explained the highest percentage of the variance in MoCA scores. None of the single-task gait metrics presented significant differences between groups.

**Conclusions:**

Our preliminary results show that the newly developed gait analysis solution based on foot-worn inertial sensors is a pertinent tool to evaluate gait metrics affected by the cognitive status of older adults relying on single- and dual-task gait assessments. Further evaluation with a larger and more diverse group is required to establish system feasibility and reliability in clinical practice.

**Trial registration:**

ClinicalTrials.gov (identifier: NCT04587895)

## Background

Despite the advances in geriatric medicine, cognitive disorders remain a critical aspect of aging. When cognitive decline exceeds what is expected due to the normal aging process, the decline is diagnosed based on severity as Mild Cognitive Impairment (MCI) or dementia [1, 2]. In MCI, cognitive decline is greater than expected for age, but the ability to perform everyday activities is still preserved [1]. Dementia is established when the loss in cognitive functioning is significant enough to interfere with a person’s functional independence [2]. People with MCI have a higher risk of conversion to dementia. Studies suggest that 8 to 15% of the MCI cases progress to dementia each year [3]. Dementia is a major cause of disability and dependency causing a significant social and economic impact worldwide [4]. Around 50 million people worldwide are living with dementia, and its prevalence is expected to triple in the next 30 years resulting from the rapid aging of the population [4].

Early diagnosis of cognitive impairment remains the most promising strategy to prevent subsequent disability and improve prognosis through early intervention [5, 6]. Traditional approaches for the screening of cognitive impairment are based on paper tests, such as the Montreal Cognitive Assessment (MoCA) [7]. These tools require time investment involving a trained questioner, being unsuitable to be carried out frequently [8]. The subtlety of symptoms, the busy pace of clinical environments, and the lack of objective, robust, and fast screening tools constitute barriers to diagnosis. The detection of cognitive impairment is thus likely to be missed or delayed, and individuals threatened with cognitive impairment receive inadequate intervention [5, 9].

Although the main clinical hallmark of cognitive impairment is cognitive decline, recent studies show that motor decline can also be observed, accompanying, or even preceding the onset of cognitive impairment [10–12]. In individuals with cognitive impairment, lower gait performance is characterized by a significant reduction in gait speed, stride length, and cadence, and an increase in double support time and gait variability [13–15]. Worsening of gait performance determined through these parameters associates with global imaging markers of a “deteriorating brain” [16]. Longitudinal studies show that gait slowing precedes cognitive impairment by 7 to 12 years [11, 17], possibly serving as a predictor of cognitive decline [18]. Slow gait has also been associated with other adverse health outcomes (e.g., increased risk of falls [19, 20], frailty [21], disability [20, 22], or mortality [21]). Hence, gait speed alone might lack specificity for the detection of cognitive impairment [16, 18, 23].

Previous work in cognitive neuroscience showed that motor and cognitive functions are interrelated and that intact cognition (in particular, executive functions) is required for competent motor control [24, 25]. The quality of walking while simultaneously performing a cognitive (attention-demanding) task (e.g., naming animals, or doing serial subtractions)—known as dual-tasking—unmasks latent gait disturbances that may only be evident under cognitive stress [10, 26]. The quality of gait under dual-task testing is affected by cognitive impairment and can distinguish MCI from preserved cognition in older adults, exposing symptoms that would otherwise be invisible [27]. Muir, S. et al. [28] assessed gait in cognitively normal, MCI, and dementia groups, and concluded that gait performance under single-task could not provide a significant distinction between groups, whereas dual-task testing provided a significant distinction. A meta-analysis showed that several gait parameters (incl. speed, stride length, and stride time) could discriminate MCI from healthy controls under the single-task condition, but the dual-task assessment increased their discriminative power [23]. The change in gait performance from a single- to a dual-task scenario (known as dual-task effect or cost) could differentiate people with cognitive impairment [29, 30]. A recent meta-analysis concluded that dual-task effects on gait speed are highly sensitive for the detection of MCI [27]. Taken together, the findings from previous studies suggest that dual-task testing might serve as a sensitive measure to improve the early detection of cognitive impairment [10, 23, 31, 32].

Although many studies support the use of dual tasks to improve the detection of cognitive impairment, some studies show contradictory results. For instance, in [33] and in [34] dual tasking could not distinguish people with preserved cognitive skills from those with MCI. Studies differed on the protocols used, including the cognitive tasks and the gait evaluation methods. However, not all cognitive tasks may be adequate to stress cognition in older adults [23, 28] and not all gait evaluation methods may be adequate for a robust evaluation of gait metrics [35].

Conventional gait and balance tests based on the use of a stopwatch only allow the assessment of a limited set of metrics and may not be sensitive to the subtle motor deficits associated with MCI [35, 36]. Some studies suggested that evaluating gait by a single characteristic may not be powerful enough to predict cognitive status and that a multidimensional gait analysis approach is required [37, 38] that should be complemented by measures of cognition [16].

The technology used for an objective and multidimensional assessment of gait—e.g., instrumented walkways, and optical motion capture systems—is expensive and requires dedicated spaces not being ideal for routine screening within clinical settings [35]. Additionally, they have a limited coverage area [35], which may potentially compromise the assessment of metrics like gait variability due to insufficient walking distance [23]. Alternative instruments are thus needed to translate research into clinical practice. Wearable technology based on inertial sensors offer a low-cost alternative for the assessment of gait, allowing gait parameters to be captured both at the clinic or in free-living conditions [35]. Due to advances in data analytics, wearables are now widely proposed as practical tools to aid the diagnosis and treatment of a range of neurological disorders [39]. However, the ability of wearables and their associated algorithms to detect cognitive impairment has yet to be established [35, 40].

Inertial sensors placed on the trunk have been used in the past to evaluate gait and differentiate cognitive status. Stride frequency, gait regularity, gait speed, stride length, duration of swing, stance, and double support phases were some of the metrics analyzed [15, 36, 41–44]. Recently, our research group proposed a new approach for the gait analysis resorting to inertial sensors placed on the shoes [45, 46]. In [45] and in [46] we proposed a new data analysis approach that is independent of the orientation of the sensor on the shoes, and that allows the evaluation of an extensive set of gait metrics and foot—heel and toe—trajectories.

This pilot study aims to investigate the potential of the newly developed foot-worn inertial sensor-based gait analysis solution to capture and differentiate gait performance in older adults with and without cognitive impairment. We hypothesized that (i) people with cognitive impairment would demonstrate more significant dual-task effects on gait metrics than older adults without cognitive impairment, (ii) the effects would be visible in multiple gait parameters, (iii) dual-task gait metrics and dual-task costs would better differentiate between groups than single-task gait metrics, and (iv) multiple gait parameters would be associated with the global cognitive performance. This paper constitutes the first step toward developing a screening tool for cognitive impairment that does not heavily rely on cognitive testing.

## Methods

This study provides a secondary analysis of anonymized baseline data (pre-measurements) collected under the scope of a Randomized Controlled Pilot Trial. The trial was originally designed to assess the feasibility of a newly developed exergame in patients with mobility impairment. The full details of the trial can be found on ClinicalTrials.gov (identifier: NCT04587895). In this study, we used the baseline data from the trial to investigate the potential of foot-worn inertial sensors and gait analysis to assess gait metrics that are affected by the cognitive status of the participants. We adhere to guidelines to report the results of these cross-sectional data [47].

### Participants and procedure

Participants were recruited through local contact persons at the physiotherapy clinic Physio SPArtos (Interlaken, Bern, Switzerland) and public advertisements in local newspapers in the surroundings of Interlaken (Bern, Switzerland). The study took place on Physio SPArtos’ premises.

Participants were included if they (i) were aged 60+ years old, (ii) lived independently, in a residency dwelling, or with care, (iii) could stand straight for at least 10 minutes without aids, (iv) had visual acuity with correction sufficient to work on a TV screen, and (v) had Short Physical Performance Battery (SPPB) test score below 10 as an indication of impaired mobility [48, 49].

Subjects manifesting one or more of the following criteria were excluded from the study: (i) Mobility impairments that did not allow to play the exergame [50]; (ii) Severe cognitive impairment (below the 1st percentile according to [51]); (iii) Acute or unstable chronic diseases (e.g., recent cardiac infarction, uncontrolled high blood pressure or cardiovascular disease, uncontrolled diabetes); (iv) Orthopedic or neurological diseases that inhibited exergame training; (v) Rapidly progressive or terminal illness; (vi) Insufficient knowledge of German; (vii) Chronic respiratory disease; (viii) Condition or therapy that weakens the immune system; (ix) Cancer; and (x) Serious obesity (Body Mass Index (BMI) $>40$ kg/m^2^).

Baseline data were acquired in a single visit to the study site, lasting around 1 to 1.5 hours. All eligible participants were assessed for demographics and medical history, cognitive tests, physical tests, and gait tests under single- and dual-task conditions.

The study was approved by the Institutional Ethics Committee of ETH Zurich (registration: 2020-00578) and followed the ethical code for research with human beings as stated by the Declaration of Helsinki. Written informed consent was obtained from all participants. No compensation for participation was given.

### Demographic data and medical history

A health questionnaire was applied to describe general information about the participants, including age, sex, weight, height, and years of education. The questionnaire also covered general health, medical history, and physical activity, including self-reported variables such as pain, medical conditions, level of physical activity, fear of falling, past falls, and walking aids. Body mass index (BMI, in Kg/m^2^) was calculated from weight and height.

### Cognitive tests

General cognition was assessed using the Montreal Cognitive Assessment (MoCA) [52]. MoCA is the neuropsychological test recommended for the screening of MCI [7]. It is a paper-and-pencil test and has a maximum score of 30 points. It takes between 10 and 20 minutes to administer [53] and evaluates different cognitive domains such as executive function, memory, language, visuospatial ability, orientation, attention, concentration, and working memory [52]. The score is corrected for low education ($\le 12$ years) by adding an extra point [52]. Although the initially proposed cut-off of $<26$ has shown good sensitivity and specificity for detecting MCI [52], the cut-off score of $<24$ resulted in better classification rates in German-speaking, Swiss, older adults [54].

Specific cognitive domains related to executive functions were assessed using (i) the Trail Making Test (TMT) Part A—for general information processing speed—and Part B—for mental flexibility [55]; (ii) the Color Word Interference Test (CWIT), to evaluate response inhibition and interference [56], and (iii) the Digit Backward Task of the Wechsler Memory Scale-Revised (WMSR), to evaluate working memory [57].

### Physical tests

The Short Physical Performance Battery (SPPB) was applied to evaluate functional physical performance. SPPB consists of three subtests: (i) maintaining balance with the feet in tandem, semi-tandem, and side-by-side, (ii) walking 4 meters, and (iii) rising and sitting five times on a chair without the help of the upper limbs. The test score is obtained by adding the scores of the subtests, resulting in values between 0 (worst performance) and 12 (best performance) [58, 59]. The SPPB has been used to characterize functional capacity in older adults with and without cognitive impairment [60], showing little change in relation to aspects such as culture, language, or educational level [61, 62]. An SPPB score lower than 10 is predictive of all-cause mortality [63] and is usually accepted as a threshold for functional impairment [64]. All participants in this study had SPPB scores $<10$.

Additionally, the 1-Minute Sit to Stand Test (MSTS) was employed to measure exercise capacity. In this test, the participants cross their arms over the chest and rise from a chair as often as possible in one minute. The score is the number of complete sit-to-stand cycles performed in 1 min [65].

### Gait assessment

Participants walked at their preferred speed (single task) and while performing a secondary cognitive task (dual task), along a straight 20m distance path. In the dual-task condition, participants had to count backward in steps of seven from a random given number between 200 and 250 while they were walking. This cognitive task has been previously recommended to stress cognition in dual-task tests [23, 27, 28]. Participants had to count out loud for the test to be considered valid. No prioritization instruction was given. The order of the tasks was maintained for all the participants, starting with the single task. The wearable devices used to capture walking data were equipped with a tri-axial gyroscope and a tri-axial accelerometer (Bosch BMI160) and were developed in our lab [45]. They were connected via Bluetooth to the computer and were used to capture raw acceleration and angular rate, which were recorded to a text file. Inertial sensors were placed on the shoes near the instep region (as described in [45]). Sensor data were sampled at a frequency of 100 Hz. Participants wore their own, low-heeled, regular shoes.

The extraction of gait metrics was performed using the method described in [45], which was implemented in Python 3. Very briefly, the method resorts to an orientation-invariant approach that comprises the steps of detecting zero velocity intervals (using the angular rate energy detector), estimating sensor orientation (using the Euston Complementary Filter), obtaining displacements (using direct and reverse integration), detecting gait events (from low-pass filtered acceleration magnitude and vertical acceleration), and, finally, estimating spatiotemporal gait metrics [45]. Further details about the method can be found in [45]. Data processing was performed offline, taking into account all the data available in recorded text files. After excluding the first and last two strides to focus the analysis on steady-state walking [25], the following metrics were extracted per stride: Stride time (s), Swing time (s), Stance time (s), Swing ratio (%), Stance ratio (%), Foot flat ratio (%), Pushing ratio (%), Loading ratio (%), Stride length (m), Gait speed (m/s), Strike angle (degrees), Liftoff angle (degrees), and Minimum toe clearance (cm). The stance ratio was calculated as a proportion of stride time. The ratios of foot flat (time during which the foot is in full contact with the ground), pushing (the time from foot flat to toe-off), and loading (the time from heel strike to foot flat) were calculated as proportions of stance time. The liftoff angle was the angle made by the foot at foot-off, relative to the ground [45]; the strike angle was determined at heel-strike. The angles were signed and were negative for the strike. Minimum toe clearance was defined as the minimum height of the toes during the swing phase of walking, as presented in [45].

The method described in [46], resorting to a deep learning approach, was used to estimate heel and toe trajectories, from which the corresponding 3D path lengths were calculated. The method used a stacked bidirectional long short-term memory (LSTM) recurrent neural network implemented in Python 3 using conda TensorFlow 2.3 to estimate trajectories from acceleration and angular rate data. To describe the variability of the trajectories, we first resampled the strides by expressing their time in percentual terms (between 0 and 100%). Then, we calculated the mean absolute deviation (MAD) for each stride, trajectory (heel and toe), and component (forward, sideward, and vertical) according to Eq. (1):

1 $$\begin{aligned} MAD = \frac{1}{n} \sum \limits _{i=1}^{n} |x_i - \bar{x_i}| \end{aligned}$$

where *n* is the number of samples in a stride, $x_i$ is the value of the trajectory at sample *i*, and $\bar{x_i}$ is the average value of all strides at sample *i*.

For each gait test, we reported the average of the metrics along all the strides (including right and left sides). We have also calculated the variability of the metrics, quantified using the coefficient of variation ($CV [\%] = 100 \times (\text {standard deviation} / \text {mean}$)). The symmetry index was used to assess asymmetry between left and right gait parameters using the formula $SI [\%] = 100 \times |\text {left side} - \text {right side}| / (0.5 \times (\text {left side} + \text {right side}))$. Moreover, for each subject and each gait parameter, we calculated the dual-task effect (DTE) of walking as a percentage of loss (or gain) relative to the single-task walking performance, according to the formula $DTE [\%] = 100 \times (\text {dual-task score} - \text {single-task score}) / \text {single-task score}$. A positive DTE corresponded to an increase in the value of the gait parameter under dual-tasking, whereas a negative DTE corresponded to a decrease.

### Statistical analysis

Participants were stratified into two groups, according to their MoCA scores, based on the cut-offs proposed by [54] and [66], as follows: they were considered controls with normal cognition if they had MoCA score $\ge 24$ (control group); they were considered to have cognitive impairment (CI) when MoCA score $< 24$ (CI group). In the CI group, two persons could not finish the TMT B test, as it took too long. For this reason, their values were replaced by the maximum.

The statistical analysis was performed using SPSS 27.0 for Mac OS (SPSS Inc, Chicago, Illinois, USA) and Python 3 (using the packages pingouin v0.4.0, StatsModels v0.13.0, SciPy v1.7.1, and NumPy v1.21.5). A *p*-value of less than 0.05 indicated statistical significance.

Demographic and medical characteristics were summarized using either means and standard deviations, or frequencies and percentages, as appropriate. Comparisons between groups were made using the Independent Samples T-test or the Mann-Whitney U test for continuous variables, and Pearson’s chi-square test ($\chi ^2$) or Fisher’s exact test for categorical variables. The Mann-Whitney U test was used when data were not normally distributed or when outliers were present. Fisher’s exact test was used when at least one cell of the contingency table had an expected frequency of less than 5. Asymptotic significance was reported for the Mann-Whitney U. The assumption of homogeneity of variance was checked using Levene’s test; when $p<0.05$, equal variances could not be assumed, and the Welch’s t-test was computed instead of the standard Independent Samples T-test.

To assess differences between single-task and dual-task conditions within each group (CI and control), we used Paired Samples T-tests or Wilcoxon signed-rank tests. Wilcoxon signed-rank tests were used when paired data differences were not normally distributed or when outliers were present. Asymptotic significance was reported for Wilcoxon.

Group differences in gait parameters were examined using the Independent Samples T-test or the Mann-Whitney U test. Interaction between gait condition (single-task *vs* dual-task) and group (control group *vs* CI group) was assessed by comparing the DTEs between groups.

Data were tested for normality using the Shapiro-Wilk test. Outliers were detected using box plots.

To examine how well gait performance could distinguish between CI and control groups, we fitted logistic regression models. Separate models were built for each gait parameter that differed significantly between groups in previous steps. Subsequently, receiver operating characteristic (ROC) curves were constructed, and the sensitivity, specificity, and area under the curve (AUC) were estimated. The optimal cut-point was considered the point of the ROC curve closest to the upper left corner (0,1). Mathematically, the optimal sensitivity and specificity were defined as those yielding the minimum value of $(1-sensitivity)^2 + (1-specificity)^2$ [67]. AUC values were interpreted as follows: $AUC = 0.5$, no discrimination; $0.5< AUC < 0.7$, poor discrimination; $0.7 \le AUC < 0.8$, acceptable discrimination; $0.8 \le AUC < 0.9$, excellent discrimination; and $AUC \ge 0.9$, outstanding discrimination [68].

Gait metrics that differed significantly between groups were included as predictors of MoCA scores (global cognition) in bivariate linear regression models to test their association with a progressive score. To obtain the best set of variables associated with MoCA scores, we used a stepwise forward approach, successively entering variables with $p<0.05$ and removing those with $p>0.1$. The stepwise regression analysis was repeated controlling for age, sex, and education.

## Results

A total of 29 participants (average age $81.24 \pm 8.35$ years old, 48.3% women) were enrolled in the study, from which 13 were considered healthy controls and 16 had cognitive impairment according to MoCA.

### Descriptive statistics

Table 1 presents the descriptive statistics for the demographic and medical data, stratified by cognitive status (healthy controls *vs* cognitively impaired). Participants differed in global cognition (MoCA score), TMT A and B scores, and CWIT inhibition times. The differences in MoCA scores between groups were expected as they defined membership in each group. The times of TMT A, TMT B, and CWIT inhibition were significantly higher in the group with MoCA score $<24$, indicating worse performance on these tests. The remaining variables, including demographic characteristics (e.g., age, BMI, education, etc.) and physical test metrics (SPPB and MSTS scores) presented no significant differences between the two groups. For this reason, these variables were not accounted as covariates in the subsequent steps of the statistical analysis.

**Table 1:** Demographic and clinical characteristics of study participants stratified by cognitive status

Participant Characteristics	Control Group (*n*=13)	CI group (*n*=16)	*p*-value
Age [years]	78.5 ± 9.2 (64-93)	83.5 ± 7.1 (68-96)	0.107
BMI [Kg/m^2^]	28.0 ± 4.5 (21.3-33.6)$^\dagger$	25.3 ± 2.5 (20.9-31.0)	0.114
Weight [Kg]	80.5 ± 15.1 (58-110)	71.4 ± 13.2 (52-95)	0.095
Height [cm]	169.7 ± 6.4 (162-182)$^\dagger$	167.6 ± 11.6 (148-184)	0.529
Education [years]	13.3 ± 4.2 (8-24)	11.5 ± 1.9 (9-15)	0.232
Female, n (%)	8 (61.5)	6 (37.5)	0.198
Hearing problems, n (%)	7 (53.8)	6 (37.5)	0.379
Vision problems, n (%)	7 (53.8)	7 (43.8)	0.588
Dizziness, n (%)	3 (23.1)	8 (50.0)	0.249
Gait problems, n (%)	8 (61.5)	10 (62.5)	1.000
**Fear of falling**			
Never, n (%)	3 (23.1)	3 (18.8)	0.755
Sometimes, n (%)	7 (53.8)	9 (56.3)	
Often, n (%)	1 (7.7)	3 (18.8)	
Always, n (%)	2 (15.4)	1 (6.3)	
**Number of falls in the last 6 months**			
Zero, n (%)	8 (61.5)	9 (56.3)	1.000
One, n (%)	2 (15.4)	3 (18.8)	
More than one, n (%)	3 (23.1)	4 (25.0)	
**Pain**			
No pain, n (%)	1 (7.7)	2 (12.5)	0.869
Less often than daily, n (%)	6 (46.2)	9 (56.3)	
Daily, n (%)	6 (46.2)	5 (31.3)	
**Pain severity**			
Low, n (%)	6 (50.0)	6 (42.9)	0.640
Medium, n (%)	4 (33.3)	7 (50.0)	
Sometimes unbearable, n (%)	2 (16.7)	1 (7.1)	
**Walking aids**			
None, n (%)	8 (61.5)	5 (31.3)	0.275
Cane, n (%)	2 (15.4)	4 (25.0)	
Walker, n (%)	3 (23.1)	7 (43.8)	
**Physical activity**			
>3 times/week, n (%)	6 (46.2)	9 (56.3)	0.387
1-3 times/week, n (%)	7 (53.8)	5 (31.3)	
1 time/week, n (%)	0 (0.0)	2 (12.5)	
Never, n (%)	0 (0.0)	0 (0.0)	
**Cognitive tests**			
MoCA Score	25.7 ± 1.8 (24-30)	21.0 ± 1.6 (17-23)	<0.001*
TMT A Time [s]	51.1 ± 12.6 (30.5-68.8)	77.8 ± 32.5 (42.3-155.0)	0.016*
TMT B Time [s]	147.5 ± 74.0 (55.1-340.8)	264.4 ± 99.9 (91.4-386.6)	0.002*
CWIT inhibition time [s]	76.0 ± 24.2 (56.4-136.7)	110.3 ± 40.6 (59.5-187.5)	0.010*
WMSR Score	5.7 ± 1.2 (4-8)	4.9 ± 1.2 (3-7)	0.078
**Physical tests**			
SPPB Score	7.9 ± 1.3 (5-9)	7.0 ± 1.6 (4-9)	0.076
MSTS Nr of repetitions	14.3 ± 8.5 (0-26)	14.9 ± 6.2 (5-32)	0.742

Data are mean values ± standard deviation (range) or the number of participants per category (absolute and relative frequency) when indicated. Group differences were evaluated using the Independent Samples T-test, Mann-Whitney U test, Pearson’s chi-square, or Fisher’s exact test

*BMI* body mass index, *MoCA* Montreal cognitive assessment, *TMT* trail making test, *CWIT* color word interference test, *WMSR* the digit backward task of the Wechsler memory scale-revised, *SPPB* short physical performance battery, *MSTS* 1-minute sit to stand test

*$p<0.05$, *p*-values are two-tailed significance and bold values indicate significance

$^\dagger$Missing data for this variable in one individual in this group

### Differences in gait between groups

The differences in average temporal gait metrics between groups—control *vs* CI group—and within groups—single- *vs* dual-task conditions—are summarized in Table 2. Compared to the single-task condition, walking under dual-task led to significant changes in all temporal gait metrics in the CI group. The largest effect was observed on the foot flat ratio in the CI group, where the addition of the dual task increased the foot flat ratio by 21.7%. Accordingly, the addition of the dual task decreased the pushing ratio by 9.7% and the loading ratio by 9.9% in the CI group. The average stride time increased with the addition of the cognitive task, as did the swing time and stance time. However, the increase in stance time (of 17.0%) was higher than the increase in swing time (8.5%), which was reflected as an increase in stance ratio and a decrease in swing ratio. Similar effects were observed in the control group, but these effects were less pronounced. For instance, the addition of the dual task only increased foot flat ratio by 8.0% in the control group (versus 21.7% in the CI group). Not all temporal gait metrics in the control group were significantly influenced by the dual-task condition.

None of the single-task or dual-task average temporal gait metrics alone presented significant differences between groups, except dual-task swing time whose average was higher in the CI group. The DTE on stride time, swing time, stance time, foot flat ratio, and pushing ratio differed significantly between groups.

**Table 2:** Comparison of average temporal gait parameters within groups (single- *vs* dual-task) and between groups (control *vs* CI group), including dual-task effects (DTEs)

Gait metric	Control group (*n*=13)	CI group (*n*=16)	Statistic	*p*-value
Stride time DTE (%)	6.49 ± 6.63	14.28 ± 10.69	T = -2.291	0.03*
ST stride time (s)	1.15 ± 0.16	1.18 ± 0.19	U = 98.5	0.809
DT stride time (s)	1.22 ± 0.21	1.34 ± 0.23	U = 64.0	0.079
	**T = -3.424;** *p* = 0.005*	**T = -4.804; ** *p* < 0.001*		
Swing time DTE (%)	2.47 ± 6.45	8.52 ± 7.55	U = 56.0	0.035*
ST swing time (s)	0.35 ± 0.04	0.38 ± 0.05	U = 72.0	0.160
DT swing time (s)	0.36 ± 0.05	0.41 ± 0.06	U = 55.0	0.032*
	W = 25.5; *p* = 0.162	**T = -4.175; ** *p* < 0.001*		
Stance time DTE (%)	8.31 ± 7.4	16.96 ± 12.36	T = -2.216	0.035*
ST stance time (s)	0.79 ± 0.13	0.8 ± 0.14	U = 106.5	0.913
DT stance time (s)	0.86 ± 0.17	0.93 ± 0.18	U = 74.5	0.196
	**W = 3.0; ** *p* = 0.003*	**W = 5.0; ** *p* = 0.001*		
Swing ratio DTE (%)	-3.49 ± 3.12	-4.64 ± 3.74	T = 0.881	0.386
ST swing ratio (%)	31.06 ± 2.01	32.36 ± 1.91	T = -1.778	0.087
DT swing ratio (%)	29.98 ± 2.18	30.88 ± 2.44	U = 71.0	0.148
	**T = 4.114; ** *p* = 0.001*	**T = 5.354; ** *p* < 0.001*		
Stance ratio DTE (%)	1.57 ± 1.37	2.18 ± 1.58	T = -1.079	0.290
ST stance ratio (%)	68.94 ± 2.01	67.64 ± 1.91	T = 1.778	0.087
DT stance ratio (%)	70.02 ± 2.18	69.12 ± 2.44	U = 137.0	0.148
	**T = -4.114; ** *p* = 0.001*	**T = -5.354; ***p*< 0.001*		
Foot flat ratio DTE (%)	7.99 ± 7.92	21.74 ± 18.37	T = -2.7	0.013*
ST foot flat ratio (%)	37.67 ± 6.25	34.56 ± 6.73	U = 137.0	0.148
DT foot flat ratio (%)	40.76 ± 7.67	41.29 ± 5.77	U = 108.0	0.861
	**T = -3.637; ** *p* = 0.003*	**T = -5.208; ** *p* < 0.001*		
Pushing ratio DTE (%)	-2.50 ± 6.45	-9.70 ± 8.44	U = 159.0	0.016*
ST pushing ratio (%)	31.97 ± 3.25	34.09 ± 4.88	T = -1.342	0.191
DT pushing ratio (%)	31.16 ± 3.67	30.59 ± 3.84	U = 117.0	0.569
	W = 23.0; *p* = 0.116	**W = 3.0; ** *p* < 0.001*		
Loading ratio DTE (%)	-8.18 ± 6.3	-9.89 ± 10.47	U = 130.0	0.254
ST loading ratio (%)	30.36 ± 5.0	31.35 ± 5.5	U = 98.0	0.792
DT loading ratio (%)	28.07 ± 6.02	28.12 ± 5.08	U = 106.5	0.913
	**W = 2.0; ** *p* = 0.002*	**W = 13.0; ** *p* = 0.004*		

Data are mean values ± standard deviation. Between-group differences were evaluated using the Independent Samples T-test (T) or the Mann-Whitney U test (U), as appropriate. Differences between single- and dual-task measures were evaluated using Paired Samples T-tests (T) or Wilcoxon signed-rank tests (W), as appropriate

*DTE* dual-task effect, *ST* single-task, *DT* dual-task

*$p<0.05$, *p*-values are two-tailed significance and bold values indicate significance

Differences in average spatial gait metrics are shown in Table 3. As observed in temporal metrics, all spatial gait metrics were significantly affected by the addition of the cognitive task in the CI group. The addition of the dual task led to a significant decrease in all average spatial metrics, being the largest effects observed on strike angle (25.2%) and gait speed (24.4%) in the CI group. In the control group, only minimum toe clearance was not significantly affected by the dual-task condition. None of the single-task or dual-task average spatial gait metrics presented significant differences between groups. The DTE on gait speed, liftoff angle, minimum toe clearance, and heel 3D path length differed significantly between groups.

**Table 3:** Comparison of average spatial gait parameters within groups (single- *vs* dual-task) and between groups (control *vs* CI group), including dual-task effects (DTEs)

Gait metric	Control group (*n*=13)	CI group (*n*=16)	Statistic	*p*-value
Stride length DTE (%)	-9.07 ± 7.48	-15.33 ± 9.14	U = 144.0	0.079
ST stride length (m)	1.10 ± 0.19	1.06 ± 0.18	U = 118.5	0.525
DT stride length (m)	1.00 ± 0.21	0.91 ± 0.2	U = 131.0	0.236
	**T = 4.803; ** *p* < 0.001*	**T = 8.160; ** *p* < 0.001*		
Speed DTE (%)	-13.94 ± 9.79	-24.39 ± 13.56	T = 2.325	0.028*
ST speed (m/s)	0.98 ± 0.24	0.93 ± 0.25	T = 0.560	0.580
DT speed (m/s)	0.85 ± 0.23	0.71 ± 0.21	T = 1.743	0.093
	**W = 3.0; ** *p* = 0.003*	**T = 6.853; ** *p* < 0.001*		
Strike angle DTE (%)	-17.04 ± 20.04	-25.24 ± 30.65	U = 129.0	0.273
ST strike angle ($^{\circ }$)	-20.20 ± 7.94	-21.13 ± 7.09	T = 0.335	0.74
DT strike angle ($^{\circ }$)	-17.48 ± 8.21	-17.14 ± 7.72	U = 105.0	0.965
	**T = -4.210; ** *p* = 0.001*	**T = -7.123; ** *p* < 0.001*		
Liftoff angle DTE (%)	-6.05 ± 5.28	-13.56 ± 9.58	U = 153.0	0.032*
ST liftoff angle ($^{\circ }$)	53.69 ± 8.77	50.38 ± 7.29	T = 1.110	0.277
DT liftoff angle ($^{\circ }$)	50.48 ± 8.93	43.87 ± 9.31	T = 1.936	0.063
	**W = 5.0; ** *p* = 0.005*	**T = 6.598; ** *p* < 0.001*		
Min toe clearance DTE (%)	-6.35 ± 18.26	-18.36 ± 22.54	U = 149.0	0.048*
ST min toe clearance (cm)	1.14 ± 0.63	1.60 ± 1.1	U = 65.0	0.087
DT min toe clearance (cm)	1.07 ± 0.62	1.25 ± 0.8	U = 90.0	0.539
	T = 1.238; *p* = 0.239	**W = 12.0; ** *p* = 0.004*		
Heel 3D path len. DTE (%)	-7.28 ± 5.85	-12.23 ± 6.21	T = 2.190	0.037*
ST heel 3D path len. (m)	1.31 ± 0.2	1.29 ± 0.21	T = 0.297	0.769
DT heel 3D path len. (m)	1.22 ± 0.2	1.13 ± 0.21	T = 1.067	0.296
	**T = 4.531; ** *p* < 0.001*	**T = 8.063; ** *p* < 0.001*		
Toe 3D path len. DTE (%)	-9.06 ± 7.23	-14.6 ± 7.63	T = 1.991	0.057
ST toe 3D path len. (m)	1.21 ± 0.23	1.19 ± 0.24	T = 0.277	0.784
DT toe 3D path len. (m)	1.11 ± 0.24	1.02 ± 0.24	U = 125.0	0.357
	**T = 4.689; ** *p* < 0.001*	**W = 0.0; ** *p* < 0.001*		

Data are mean values ± standard deviation. Between-group differences were evaluated using the Independent Samples T-test (T) or the Mann-Whitney U test (U), as appropriate. Differences between single- and dual-task measures were evaluated using Paired Samples T-tests (T) or Wilcoxon signed-rank tests (W), as appropriate

*DTE* dual-task effect, *ST*, single-task, *DT* dual-task

*$p<0.05$, *p*-values are two-tailed significance and bold values indicate significance

**Fig. 1:**
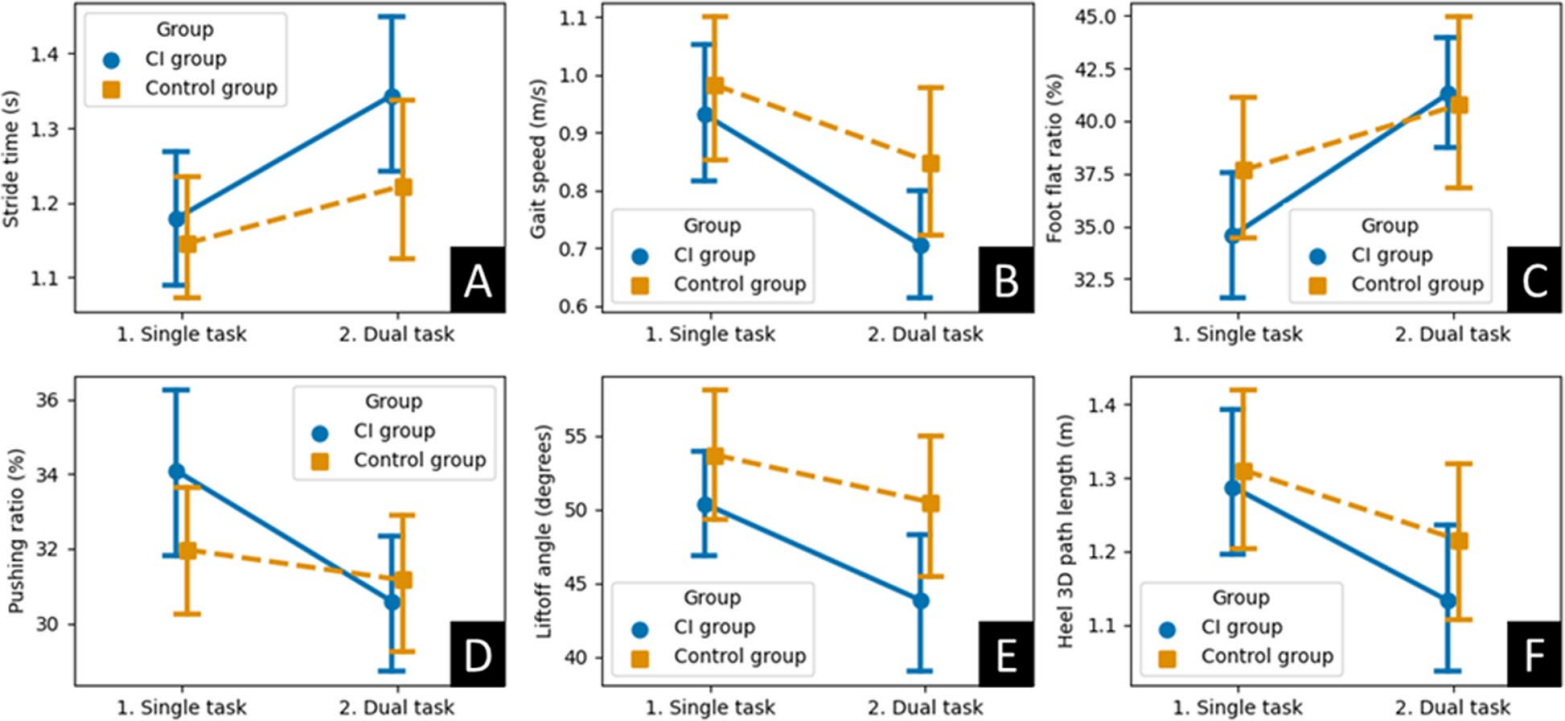
Group $\times$ task interaction for **A** stride time, in seconds, **B** gait speed, in m/s, **C** foot flat ratio, in %, **D** pushing ratio, in %, **E** liftoff angle, in degrees, and **F** heel 3D path length, in meters. Errors bars represent 95% confidence intervals

Figure 1 shows interaction plots for some of the gait metrics whose DTEs presented a statistically significant difference between groups in Tables 2 and 3. Compared to single-task, the dual-task condition had a significant effect on all gait parameters and groups presented in Fig. 1, except the pushing ratio in the control group (Table 2). The effects were more pronounced in the CI group leading to significant differences in the DTEs. In Fig. 1, the blue solid lines (CI group) had higher slopes than the orange dashed lines (control group) which denoted these effects.

Differences between groups on dual-task variability metrics are shown in Table 4. Some of the metrics presented significant differences between groups, being their averages higher in the CI group. Among temporal metrics, stride, stance, and swing times variability presented significant differences between groups, whereas none of the gait phase ratios did. Speed and liftoff angles variabilities also presented significant differences between groups, although the differences in liftoff angles variability were marginally significant. The differences in stride length variability were significant and consistent with the differences in heel and toe trajectory forward MAD, and heel and toe 3D path length variability—all of them related to an increased variability of foot trajectories in the CI group under dual-task gait testing.

**Table 4:** Comparison of dual-task variability measures between groups

Gait metric	Control group (*n*=13)	CI group (*n*=16)	Statistic	*p*-value
Stride time variability (%)	4.81 ± 4.77	7.23 ± 5.78	U = 51.0	0.020*
Swing ratio variability (%)	6.74 ± 3.86	8.43 ± 4.73	U = 67.5	0.109
Stance ratio variability (%)	2.81 ± 1.42	3.63 ± 1.63	U = 65.0	0.087
Swing time variability (%)	6.87 ± 3.45	10.81 ± 8.02	U = 49.0	0.016*
Stance time variability (%)	6.62 ± 7.03	8.91 ± 6.37	U = 56.5	0.037*
Foot flat ratio variability (%)	11.75 ± 4.67	14.71 ± 5.32	T = -1.570	0.128
Pushing ratio variability (%)	9.80 ± 4.29	14.45 ± 7.38	U = 67.0	0.105
Loading ratio variability (%)	12.90 ± 5.45	16.81 ± 7.71	U = 74.0	0.188
Speed variability (%)	7.91 ± 4.81	10.76 ± 4.33	U = 56.0	0.035*
Stride length variability (%)	5.62 ± 3.57	7.63 ± 2.97	U = 50.0	0.018*
Strike angle variability (%)	-29.18 ± 38.95	8.45 ± 104.41	U = 106.0	0.930
Liftoff angle variability (%)	8.57 ± 4.27	13.00 ± 7.09	U = 58.0	0.044*
Min toe clearance variability (%)	42.57 ± 13.87	44.56 ± 20.68	T = -0.297	0.769
Heel trajectory forward MAD (mm)	25.22 ± 8.94	32.23 ± 8.54	U = 53.0	0.025*
Heel trajectory sideward MAD (mm)	2.65 ± 0.57	2.47 ± 0.40	T = 1.007	0.323
Heel trajectory vertical MAD (mm)	5.61 ± 2.02	6.47 ± 1.93	U = 80.0	0.293
Toe trajectory forward MAD (mm)	25.28 ± 9.29	32.03 ± 7.85	U = 53.0	0.025*
Toe trajectory sideward MAD (mm)	2.80 ± 0.89	2.57 ± 0.82	U = 117.0	0.569
Toe trajectory vertical MAD (mm)	2.41 ± 0.82	2.69 ± 0.61	U = 77.0	0.236
Heel 3D path length variability (%)	4.70 ± 3.13	6.47 ± 3.86	U = 59.0	0.048*
Toe 3D path length variability (%)	5.55 ± 3.28	7.41 ± 2.97	U = 53.0	0.025*

Data are mean values ± standard deviation. Group differences were evaluated using the Independent Samples T-test (T) or the Mann-Whitney U test (U), as appropriate

*MAD* mean absolute deviation

*$p<0.05$, *p*-values are two-tailed significance and bold values indicate significance

Table 5 shows the differences between groups on dual-task asymmetry metrics. Only two asymmetry measures (on loading ratio and liftoff angles) presented significant differences between groups, although these differences were marginally significant. The average values of loading ratio asymmetry and liftoff angles asymmetry were higher in the CI group.

**Table 5:** Comparison of dual-task asymmetry measures between groups

Gait metric	Control group (*n*=13)	CI group (*n*=16)	Statistic	*p*-value
Stride time asymmetry (%)	0.35 ± 0.32	0.52 ± 0.65	U = 92.5	0.614
Stance ratio asymmetry (%)	1.74 ± 1.16	2.72 ± 2.55	U = 85.0	0.405
Swing time asymmetry (%)	3.99 ± 2.91	6.27 ± 6.09	U = 89.5	0.525
Foot flat ratio asymmetry (%)	10.01 ± 7.86	13.30 ± 10.06	T = -0.960	0.345
Pushing ratio asymmetry (%)	7.95 ± 6.43	14.06 ± 14.70	U = 77.0	0.236
Loading ratio asymmetry (%)	7.47 ± 6.96	13.19 ± 7.83	U = 59.0	0.048*
Stride length asymmetry (%)	1.57 ± 0.99	2.79 ± 2.28	U = 66.5	0.100
Speed asymmetry (%)	1.20 ± 1.02	2.59 ± 2.36	U = 61.0	0.059
Strike angle asymmetry (%)	-29.78 ± 52.78	-2.01 ± 42.12	U = 79.0	0.273
Liftoff angle asymmetry (%)	5.55 ± 5.74	11.09 ± 8.18	U = 57.0	0.039*
Min toe clearance asymmetry (%)	48.14 ± 24.12	40.22 ± 32.78	T = 0.725	0.475
Heel 3D path length asymmetry (%)	2.00 ± 1.99	3.16 ± 3.62	U = 86.0	0.430
Toe 3D path length asymmetry (%)	2.79 ± 1.98	3.53 ± 2.56	T = -0.856	0.400

Data are mean values ± standard deviation. Group differences were evaluated using the Independent Samples T-test (T) or the Mann-Whitney U test (U), as appropriate

*$p<0.05$, *p*-values are two-tailed significance and bold values indicate significance

### Prediction of cognitive impairment

Gait metrics that differed significantly between groups in the previous section were included in the ROC analysis to test their capability to accurately identify clinical groups. Table 6 shows the classification performance of each gait parameter based on ROC analysis.

**Table 6:** Classification performance based on logistic regression and ROC Analysis (*n*=29)

Gait metric	**AUC**	SE	*p*-value	Sens./Spec.
Stride time DTE (%)	0.740	0.097	0.028*	0.813 / 0.692
Foot flat ratio DTE (%)	0.760$^\dagger$	0.090	0.018*	0.625 / 0.846
Pushing ratio DTE (%)	0.764$^\dagger$	0.092	0.016*	0.750 / 0.769
Speed DTE (%)	0.707	0.096	0.059	0.625 / 0.692
Liftoff angle DTE (%)	0.736	0.094	0.032*	0.688 / 0.769
Min toe clearance DTE (%)	0.716	0.097	0.048*	0.750 / 0.615
Heel 3D path length DTE (%)	0.712	0.097	0.054	0.625 / 0.769
DT Stride time variability (%)	0.755$^\dagger$	0.095	0.020*	0.750 / 0.769
DT Stride length variability (%)	0.760$^\dagger$	0.101	0.018*	0.750 / 0.769
DT Speed variability (%)	0.731	0.101	0.035*	0.750 / 0.692
DT Liftoff angle variability (%)	0.721	0.098	0.044*	0.688 / 0.846
DT Heel trajectory forward MAD (mm)	0.745	0.097	0.025*	0.813 / 0.769
DT Toe trajectory forward MAD (mm)	0.745	0.100	0.025*	0.813 / 0.769
DT Heel 3D path length variability (%)	0.716	0.100	0.048*	0.750 / 0.692
DT Toe 3D path length variability (%)	0.745	0.098	0.025*	0.750 / 0.692
DT Loading ratio asymmetry (%)	0.716	0.102	0.048*	0.938 / 0.538
DT Liftoff angle asymmetry (%)	0.726	0.095	0.039*	0.750 / 0.692

Sensitivity (Sens.) and Specificity (Spec.) were obtained for the optimal cut-point based on ROC curve analysis. *p*-values under the null hypothesis that AUC equals 0.5

*AUC* area under the curve, *ROC* receiver-operating characteristic, *SE* standard error (under nonparametric assumption), *DTE* dual-task effect, *DT* dual-task, *MAD* mean absolute deviation

*$p<0.05$, bold values indicating significance

$$^\dagger AUC > 0.75$$

All metrics, except speed DTE and heel 3D path length DTE, had statistically significant ROC curves (AUC significantly higher than chance) and provided acceptable discrimination according to [68] ($0.7 \le AUC < 0.8$). The best combination of sensitivity and specificity was achieved with dual-task heel and toe trajectories forward MAD, both with $AUC = 0.745$ ($p<0.05$) and able to detect CI group membership with a sensitivity of 81.3% and specificity of 76.9%. The pushing ratio DTE had the highest AUC, followed by foot flat ratio DTE, dual-task stride length variability, and dual-task stride time variability, all with $AUC > 0.75$ ($p<0.05$).

### Association with global cognitive score

Gait metrics that differed significantly between groups were included as predictors of MoCA scores in multiple bivariate linear regression models to test the association of each gait metric with the global cognitive score (progressive cognitive impairment). Results are shown in Table 7.

**Table 7:** Associations between gait metrics and MoCA scores in multiple bivariate linear regression analysis (*n*=29)

Gait metric	Model Summary			Coefficients		
	R^2^	Adj. R^2^	SE	B	$\varvec{\beta }$	*p*
Stride time DTE (%)	0.193	0.163	2.660	-0.131	-0.439	0.017*
Speed DTE (%)	0.258	0.230	2.550	0.114	0.508	0.005*
Foot flat ratio DTE (%)	0.173	0.142	2.693	-0.076	-0.416	0.025*
Pushing ratio DTE (%)	0.162	0.130	2.711	0.140	0.402	0.031*
Liftoff angle DTE (%)	0.247	0.219	2.570	0.166	0.497	0.006*
Min toe clearance DTE (%)	0.166	0.135	2.705	0.056	0.407	0.028*
Heel 3D path length DTE (%)	0.244	0.216	2.575	0.222	0.494	0.006*
DT Stride time variability (%)	0.031	-0.004	2.914	-0.096	-0.177	0.357
DT Stride length variability (%)	0.097	0.064	2.813	-0.271	-0.312	0.099
DT Speed variability (%)	0.086	0.052	2.831	-0.181	-0.293	0.123
DT Liftoff angle variability (%)	0.124	0.092	2.771	-0.163	-0.353	0.061
DT Heel trajectory forward MAD (mm)	0.093	0.059	2.820	-0.096	-0.305	0.108
DT Toe trajectory forward MAD (mm)	0.064	0.029	2.865	-0.081	-0.252	0.187
DT Heel 3D path length variability (%)	0.163	0.132	2.708	-0.326	-0.404	0.030*
DT Toe 3D path length variability (%)	0.153	0.121	2.725	-0.356	-0.391	0.036*
DT Loading ratio asymmetry (%)	0.180	0.150	2.681	-0.157	-0.425	0.022*
DT Liftoff angle asymmetry (%)	0.067	0.032	2.861	-0.099	-0.258	0.177

*DTE* dual-task effect, *DT* dual-task, *MAD* mean absolute deviation, *R*^*2*^ coefficient of determination, *Adj. R*^*2*^ adjusted *R*^*2*^, *SE* standard error of the estimate, *B* unstandardized coefficients, $\beta$ standardized coefficients

*$p<0.05$, bold values indicate significance

All tested DTEs presented a significant association with MoCA scores. Additionally, dual-task heel and toe path length variabilities, and dual-task loading ratio asymmetry presented a significant negative association with MoCA. The DTE on gait speed explained the highest percentage (over 25%) of the variance in MoCA scores ($R^2 = 0.258$).

**Fig. 2:**
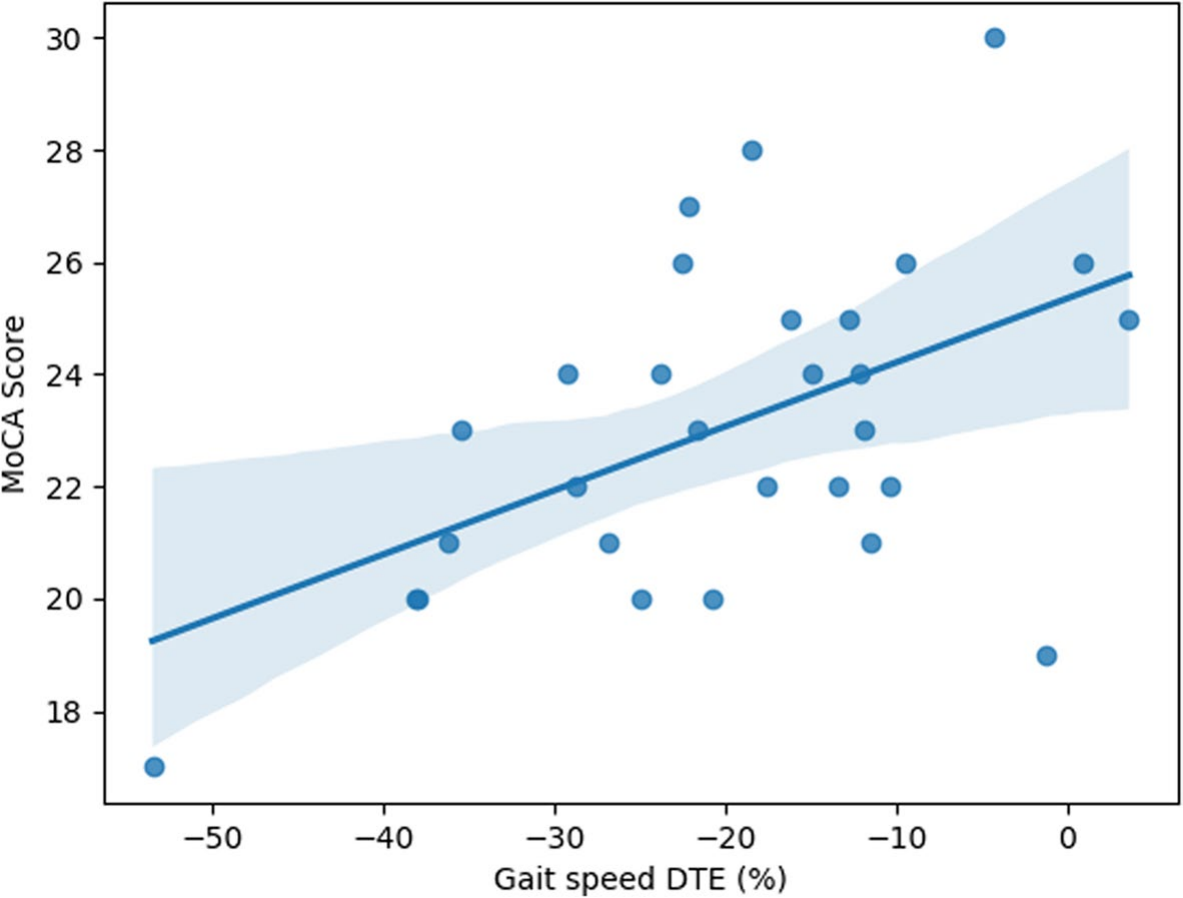
Regression model for the prediction of MoCA scores

The model resulting from the stepwise forward regression analysis only included the DTE on speed ($B=0.114$, $p=0.005$) as an independent variable. For each 10% decrease in speed DTE (%), MoCA scores decreased roughly by 1 point (Fig. 2). None of the potential covariates (i.e., age, sex, or education) were selected for the final model.

## Discussion

This study investigated the potential of a newly developed foot-worn inertial sensor-based gait analysis solution to assess gait metrics that are affected by the cognitive status of older adults using single- and dual-task gait assessments. Our contributions were the following: (i) we inspected whether the novel foot-worn inertial sensor-based gait analysis solution was able to distinguish between older adults with and without cognitive impairment; (ii) we explored differences between these two groups considering a comprehensive set of gait parameters, including dual-task metrics and dual-task costs—some of them not yet explored in the literature; (iii) we investigated which gait parameters measured by the foot-worn inertial sensors were the most relevant to assess cognitive status; and (iv) for the first time, we investigated these topics in a group of older adults with mobility limitations: a very common condition among older adults, with an estimated prevalence of 23 to 47% [69].

In line with our initial hypothesis, the results of this study showed that the CI group tended to be more influenced by the dual-task than the control group, which translated into significant differences between groups on several DTEs (Tables 2 and 3) and some dual-task variability (Table 4) and asymmetry (Table 5) metrics. Also, several of these metrics provided acceptable discrimination ability (according to the ROC analysis, Table 6) and had significant association with MoCA scores (Table 7). The signs of the regression coefficients revealed positive or negative associations as expected due to the detrimental effects of the cognitive task on gait performance. The dual-task effect on gait speed explained the highest percentage of the variance in MoCA scores ($>25\%$). As hypothesized, dual-task gait metrics and dual-task costs had better discrimination ability than single-task gait metrics. None of the single-task gait metrics presented significant differences between groups.

Overall, our preliminary results confirmed that the newly developed gait analysis solution is a pertinent tool to evaluate gait metrics that are affected by the cognitive status of older adults relying on single- and dual-task gait assessments.

### The effect of the dual task on walking

This study examined the effects of dual tasking on several gait metrics. The observed increase in stance and swing times, the decrease in swing ratio, the increase in foot flat ratio, and the decrease in all average spatial metrics—including gait speed—are consistent with a poorer gait performance due to the adoption of a more cautious gait under dual-tasking, i.e., when less attentional resources were available for walking [70]. Slower gait speed due to dual tasking was also reported previously in [71] and in [30].

Gait speeds below 1.0 m/s are commonly used to identify individuals with increased risk of falls [28], which is relevant under dual-task testing as most of the activities of daily living involve the simultaneous performance of cognitive and motor tasks [28, 72]. In our study, both mobility impaired groups had an average gait speed $<1.0$ m/s, which was further lowered in dual-task conditions and in the CI group, potentially denoting an increased fall risk under real-life situations. The fact that our participants had mobility impairment may explain the lower gait speeds even under single-task conditions.

The fact that cognitively normal older adults are also affected by the dual task is not surprising, as with aging structural changes occur in the brain and gait becomes less automatic [16, 72]. Moreover, our cognitively normal group had mobility limitations (SPPB score $< 10$). Considering that motor and cognitive functions are interrelated, and that intact cognition is required for motor control, it is expected that in the CI group the quality of walking while performing an attention-demanding task is further decreased, which may serve as an early indicator of cognitive impairment [24, 25, 27].

According to several past studies, the effect of the dual task depends on the complexity or load of the cognitive task used. The cognitive task of counting backward by sevens has been consistently reported as one of the most complex tasks, leading to greater dual-task effects than less complex tasks such as counting backward by ones, or naming animals [23, 27, 28, 71, 73, 74]. The task of counting backward used in our study seems to be appropriate and explains the high dual-task effects observed in our participants. According to [23], a high cognitive load is required in a dual-task protocol for making MCI-specific gait changes emerge.

### Differences between groups

None of the single-task gait metrics presented significant differences between groups in our study. While most of the studies agreed that dual-task gait testing could create higher differences in gait between cognitively impaired and cognitively healthy groups, some studies could still find some differences under single-task conditions. For instance, gait speed was significantly lower in patients with MCI under single-task in [37, 44, 74] and [36]. In [28] gait speed under single-task gait testing did not present significant differences between groups, but, under dual-task, the MCI group differed from the control group. Similar results were obtained for gait speed in [30, 36, 41], and [75], and for stride time variability in [30]. A recent meta-analysis showed that the differences between groups on gait speed under dual-task were more obvious than in single-task, with effects sizes of $-0.89$ and $-0.74$, respectively [27].

Muir, S. et al. [28] showed that the DTE on gait speed was higher in MCI than in the cognitively normal group. Other studies reported significant interactions between gait condition (single-task *vs* dual-tasks) and group (CI group *vs* control group) on gait speed [30, 75], stride time [29, 73], and stride time variability [30, 73], suggesting that the effect of the dual task was greater in the CI group than in the control group. In our study, the DTEs on gait speed and stride time were significantly different, which is in line with past studies’ results. In [73], however, the interaction was not significant for gait speed, which contradicts the results obtained in our study and other studies in this field. Contradictory results were also obtained in [33], in which group $\times$ condition interaction was not significant for all metrics reported, i.e., gait speed, stride time, and double support ratio. Besides having included a very low number of participants (low power), in [33] the cognitive tasks (counting backward by ones, short story recall, and a phonemic fluency task) may not have been challenging enough to cause a significant effect on gait [23] (as discussed above).

In addition to the DTE on gait speed and stride time, the DTE on foot flat ratio, liftoff angle, minimum toe clearance, and heel 3D path length presented significant differences between groups in our study. None of these metrics have been studied in the past, although they are coherent with a poorer gait performance and the adoption of a more cautious gait, well evidenced by the slower speeds and shorter displacements of the feet during swing caused by higher motor-cognitive interferences in the CI group. Metrics derived from heel and toe trajectories that provided significant differences between groups were obtained by resorting to a deep learning-based approach (as documented in [46]), which denotes the potential of these novel techniques (and metrics) and their role in the identification of cognitive impairment.

The differences between groups were also significant for several dual-task variability metrics (according to Table 4) and marginally significant for two asymmetry metrics (according to Table 5). In [28] and in [30], stride time variability under dual-task gait testing was significantly higher in the MCI group, which is consistent with our results. In [41], symmetry was significantly affected by the dual task in the MCI group, although the differences between groups were not significant. Gait variability is known to rely on higher cortical brain control, such that an increased gait variability reflects cognitive and cortical deterioration [76]. Consistently, gait variability under dual-tasking was further exacerbated in the CI group according to our results.

### Cognitive status evaluation

Most of the studies agreed that dual-task gait testing could better differentiate between groups than single-task gait tests [23]. In our study, most dual-task gait metrics and DTEs could differentiate between groups, providing an acceptable discrimination ($0.7 \le AUC < 0.8$). In [75], the discrimination ability of the dual-task gait speed was classified as fair-to-good ($AUC=0.738$) and was higher than the single-task gait speed, which was classified as poor-to-fair ($AUC=0.674$). In [29], the dual-task gait performance on gait time could distinguish subjects with subjective complaints from patients with MCI, producing an AUC value of 0.63. In [74], the DTE on gait speed differed between MCI and control groups, with the MCI group experiencing higher costs than the controls. According to [27], the DTE on gait speed provides high sensitivity for the detection of MCI. In our study, the DTE on gait speed could not discriminate between CI and control groups, although other DTE metrics could (namely, the DTE on stride time, foot flat ratio, pushing ratio, liftoff angle, and minimum toe clearance, all with $AUC > 0.70$—Table 6). In contrast, the DTE on gait speed presented a significant (positive) association with MoCA scores, explaining the highest percentage (over 25%) of the variance in MoCA scores (Table 7).

All tested dual-task variability metrics and dual-task asymmetry metrics provided acceptable discrimination, but only three of them (i.e., dual-task heel and toe 3D path length variability, and dual-task loading ratio asymmetry) presented a significant (negative) association with MoCA scores. All tested DTEs presented a significant association with global cognitive score (Table 7). Past studies have also reported significant associations of gait metrics with global cognitive scores. For instance, in [15] all gait parameters (i.e., speed, stride length, cadence, and duration of stance, swing, and double support phases) had a significant correlation with cognitive status as expressed by the Addenbrooke’s Cognitive Examination Revised (ACE-R) score. In [44], gait speed and stride length were positively correlated with ACE-R. In [77], MoCA scores were significantly correlated with gait speed and stride length.

### Study strengths and limitations

Most of the previous studies relied on conventional gait analysis instruments like instrumented walkways (e.g., in [26, 28, 37, 78], and [74]), or accelerometers placed on the lower trunk (e.g., in [36, 41–44], and [15]) to evaluate the gait of older adults with and without cognitive impairment. Although foot-worn inertial sensors and a newly developed method for the gait analysis have been employed in this study, our results concerning the assessment of cognitive status showed no inferior discrimination ability, which denotes the potential of the newly developed method. The proposed solution is convenient for use in clinical settings due to its simplicity and flexibility.

In the past, studies have barely explored the utility of DTEs to discriminate between older adults with and without cognitive impairment. In our study, the DTEs on several gait metrics provided significant differences between groups (Table 2 and Table 3), could discriminate between groups (Table 6), and presented a significant association with MoCA scores (Table 7). DTEs offer a great potential to distinguish between groups as they compare dual-task gait performance with baseline (single-task) gait performance, thereby eliminating individual baseline characteristics and representing only the effects due to the addition of the cognitive task. Other metrics, like foot flat, pushing, and loading ratios, strike and liftoff angles, minimum toe clearance, and heel and toe trajectories, have never been explored in related literature which constitutes a novel contribution of our work. Parameters that are dependent on the interrelationship between both feet (e.g., double support time) could not be evaluated in our study since inertial sensors were not temporally synced. According to past works, double support time could also be an important metric to discriminate older adults with cognitive impairment [15, 78].

Metrics known to influence gait parameters, like age, sex, or height, were not considered in the analysis of group differences, because they did not differ between groups in the descriptive analysis. Yet, other potential covariates (e.g., the size of the leg according to [37], medication effects, or others) were not considered in this study, which may affect the results. For instance, some studies have considered gait speed as a covariate to control for the influence on variability parameters [37, 76] although, according to [79], the greater variability observed in the gait of older adults may result more from loss of strength and flexibility than from their slower speeds. Nevertheless, the use of the DTEs may mitigate some of the effects of the unknown or unconsidered covariates as their influences on baseline gait data are not expected to affect the dual-task decrements, i.e., study participants are considered their own baselines [29].

The utility of discrete gait characteristics to discriminate between groups was determined by the AUC of ROC curves. AUC values were between 0.7 and 0.8, indicating that none of the separate gait parameters had a strong individual predictive power. It should be noted that the reported AUC values may not accurately represent the generalization performance of the logistic regression models, as they were not evaluated in an independent test set. Although previous studies have also reported AUC values based on logistic regression models trained on the entire dataset (e.g., [37, 40, 75, 76], or [29]), it is important to interpret the results cautiously due to the potential presence of overfitting, even though only one feature (one gait metric) was used to fit the models (less prone to overfitting). Previous works suggested that the combination of metrics in logistic regression models could provide better discrimination ability [37, 38]. Considering that we had rather few participants in this study and to further avoid the issue of overfitting, we did not combine gait metrics. On the other side, several gait metrics might be highly related and should not provide additional discrimination ability. In several studies, gait metrics were summarized by their independent factors using factor analysis, yielding 3 to 5 independent domains, like pace, rhythm, variability, asymmetry, and postural control [38, 76, 78, 80], that differed significantly between groups [76, 78]. Due to the limited size of the sample in our study, we were not able to assess the independence of the gait characteristics and reduce the dimensionality of the problem. Our results thus warrant further investigations with larger and more heterogenic groups of older adults, specifically focused on assessing more complex relationships between variables and evaluating the models on independent test sets.

Also, we could not compare our results with a strict control group, i.e., a sample without mobility impairment, although mobility impairment is known to affect gait performance [81]. We should investigate whether the dual-task assessments are or are not specific to the detection of cognitive impairment when people without mobility impairment (as determined by the SPPB scores) are included. The newly developed gait analysis solution was able to find differences between those with and without cognitive impairment in a sample with mobility limitations. Considering that mobility limitations are very common in older adults—and frequently co-exist with cognitive impairment [10, 11, 82]—it is important to ensure that the developed solution can reliably assess gait in participants with these characteristics. Further research is, however, required.

In addition to the relatively small number of participants involved (low statistical power), other study limitations should be pointed out. First, CI and control groups were stratified according to their MoCA scores—the diagnosis was not provided by the neuropsychologists. Although MoCA is considered the preferred screening tool for primary care settings, it has a sensitivity of 81-97% [7], which may not be reliable. Additionally, the order of the gait tests (single-task and dual-task) was fixed, which may have served as a limitation if individuals with cognitive impairment would fatigue more easily than those without cognitive impairment. Although gait was affected by the dual task and could discriminate between groups, the effects of walking on the cognitive task were not evaluated in this study. According to [83], the correct interpretation of the dual-task interference requires an evaluation of both tasks under single- and dual-task conditions as an effect might also be observed on the performance of the cognitive task that may support the evaluation of the cognitive status (as in [43, 75], and [29]).

### Future work

Further research is required to demonstrate the validity and reliability of gait metrics for clinical decision-making. We should acquire data from a higher number of participants, include older adults without mobility limitations, and ask neuropsychologists to provide a formal clinical diagnosis of the participants. We should explore more advanced data analysis techniques, including data-driven modeling approaches, e.g., based on machine learning, to unveil more complex relationships between variables and more complex patterns within the data. The models developed to detect cognitive impairment should be evaluated in an independent test set (never utilized in the modeling phase) to assess the generalization performance of the model. Feature selection and dimensionality reduction techniques should be applied to reduce the number of input variables and ensure their independence. We should not only evaluate gait but also evaluate the performance of the cognitive task under single- and dual-task testing to ensure a complete evaluation of the motor-cognitive interference. Additional cognitive tasks might be explored to maximize the interference of the dual-task condition and its effects on the performance of gait and cognition. Finally, we should investigate the performance and the usefulness of the newly developed methods in a real application scenario using a longitudinal study design to estimate the predictive value of cognitive decline based on gait measures.

## Conclusions

The current pilot study strengthens the current evidence that dual-task gait assessments can be an important tool for the assessment of cognitive impairment. By employing foot-worn inertial sensors and the newly developed gait analysis approach, the preliminary results of this study could capture differences in multiple gait metrics related to differences in cognitive performance. Several dual-task effect metrics were explored for the first time, revealing an increased susceptibility of the cognitive impairment group to dual-task effects when compared to the control group. Further evaluation with a larger and more diverse sample is required to establish the feasibility and reliability of the system in practical scenarios. The validation of the solution in the future could make standardized dual-task assessments and foot-worn inertial sensor-based gait analysis applicable as a fast-screening tool in primary care settings to improve the early detection of cognitive impairment.

## Data Availability

The data used in this study are available from the corresponding author on reasonable request.

## Abbreviations

MCI: Mild cognitive impairment
MoCA: Montreal Cognitive Assessment
SPPB: Short Physical Performance Battery
BMI: Body Mass Index
TMT: Trail Making Test
CWIT: Color Word Interference Test
WMSR: Wechsler Memory Scale-Revised
MSTS: 1-Minute Sit to Stand Test
MAD: Mean absolute deviation
CV: Coefficient of variation
SI: Symmetry index
DTE: Dual-task effect
CI: Cognitive impairment
ROC: Receiver operating characteristic
AUC: Area under the curve
ACE-R: Addenbrooke’s Cognitive Examination Revised
ST: Single-task
DT: Dual-task

## References

[1] Petersen RC, Negash S (2008). Mild cognitive impairment: an overview. CNS Spectrums..

[2] Hugo J, Ganguli M (2014). Dementia and Cognitive Impairment: Epidemiology, Diagnosis, and Treatment. Clin Geriatr Med..

[3] Petersen RC (2016). Mild Cognitive Impairment. Continuum (Minneapolis, Minn)..

[4] GBD 2019 Dementia Forecasting Collaborators (2022). Estimation of the global prevalence of dementia in 2019 and forecasted prevalence in 2050: an analysis for the Global Burden of Disease Study 2019. Lancet Public Health..

[5] Sabbagh MN, Boada M, Borson S, Chilukuri M, Doraiswamy PM, Dubois B, et al. Rationale for Early Diagnosis of Mild Cognitive Impairment (MCI) supported by Emerging Digital Technologies. J Prev Alzheimers Dis. 2020;1–7. 10.14283/jpad.2020.19. 10.14283/jpad.2020.19 32463068

[6] Rasmussen J, Langerman H (2019). Alzheimer’s Disease - Why We Need Early Diagnosis. Degenerative Neurol Neuromuscul Dis..

[7] Abd Razak MA, Ahmad NA, Chan YY, Mohamad Kasim N, Yusof M, Abdul Ghani MKA (2019). Validity of screening tools for dementia and mild cognitive impairment among the elderly in primary health care: a systematic review. Public Health..

[8] Matsuura T, Sakashita K, Grushnikov A, Okura F, Mitsugami I, Yagi Y (2019). Statistical Analysis of Dual-task Gait Characteristics for Cognitive Score Estimation. Sci Rep..

[9] Lang L, Clifford A, Wei L, Zhang D, Leung D, Augustine G (2017). Prevalence and determinants of undetected dementia in the community: a systematic literature review and a meta-analysis. BMJ Open..

[10] Montero-Odasso M, Verghese J, Beauchet O, Hausdorff JM (2012). Gait and Cognition: A Complementary Approach to Understanding Brain Function and the Risk of Falling. J Am Geriatr Soc..

[11] Buracchio T, Dodge H, Howieson D, Wasserman D, Kaye J (2010). The trajectory of gait speed preceding MCI. Arch Neurol..

[12] Tian Q, Chastan N, Bair WN, Resnick SM, Ferrucci L, Studenski SA (2017). The brain map of gait variability in aging, cognitive impairment and dementia-A systematic review. Neurosci Biobehav Rev..

[13] Beauchet O, Allali G, Montero-Odasso M, Sejdić E, Fantino B, Annweiler C (2014). Motor phenotype of decline in cognitive performance among community-dwellers without dementia: population-based study and meta-analysis. PLoS ONE..

[14] Beauchet O, Blumen HM, Callisaya ML, De Cock AM, Kressig RW, Srikanth V (2018). Spatiotemporal Gait Characteristics Associated with Cognitive Impairment: A Multicenter Cross-Sectional Study, the Intercontinental “Gait, cOgnitiOn & Decline” Initiative. Curr Alzheimer Res..

[15] Mulas I, Putzu V, Asoni G, Viale D, Mameli I, Pau M (2021). Clinical assessment of gait and functional mobility in Italian healthy and cognitively impaired older persons using wearable inertial sensors. Aging Clin Exp Res..

[16] Wilson J, Allcock L, Mc Ardle R, Taylor JP, Rochester L. The neural correlates of discrete gait characteristics in ageing: A Structured Review. Neurosci Biobehav Rev. 2018;100. 10.1016/j.neubiorev.2018.12.017. 10.1016/j.neubiorev.2018.12.017 PMC6565843 30552912

[17] Dumurgier J, Artaud F, Touraine C, Rouaud O, Tavernier B, Dufouil C (2017). Gait Speed and Decline in Gait Speed as Predictors of Incident Dementia. J Gerontol A Biol Sci Med Sci..

[18] Kikkert LHJ, Vuillerme N, van Campen JP, Hortobágyi T, Lamoth CJ (2016). Walking ability to predict future cognitive decline in old adults: A scoping review. Ageing Res Rev..

[19] Kyrdalen IL, Thingstad P, Sandvik L, Ormstad H (2019). Associations between gait speed and well-known fall risk factors among community-dwelling older adults. Physiother Res Int..

[20] Abellan Van Kan G, Rolland Y, Andrieu S, Bauer J, Beauchet O, Bonnefoy M (2009). Gait speed at usual pace as a predictor of adverse outcomes in community-dwelling older people an International Academy on Nutrition and Aging (IANA) Task Force. J Nutr Health Aging..

[21] Jung HW, Jang IY, Lee CK, Yu SS, Hwang JK, Jeon C (2018). Usual gait speed is associated with frailty status, institutionalization, and mortality in community-dwelling rural older adults: a longitudinal analysis of the Aging Study of Pyeongchang Rural Area. Clin Interv Aging..

[22] Miller ME, Magaziner J, Marsh AP, Fielding RA, Gill TM, King AC (2018). Gait Speed and Mobility Disability: Revisiting Meaningful Levels in Diverse Clinical Populations. J Am Geriatr Soc..

[23] Bahureksa L, Najafi B, Saleh A, Sabbagh M, Coon D, Mohler MJ (2017). The Impact of Mild Cognitive Impairment on Gait and Balance: A Systematic Review and Meta-Analysis of Studies Using Instrumented Assessment. Gerontology..

[24] Sheridan PL, Hausdorff JM (2007). The role of higher-level cognitive function in gait: executive dysfunction contributes to fall risk in Alzheimer’s disease. Dement Geriatr Cogn Disord..

[25] Beauchet O, Annweiler C, Montero-Odasso M, Fantino B, Herrmann FR, Allali G (2012). Gait control: a specific subdomain of executive function?. J NeuroEngineering Rehabil..

[26] Montero-Odasso M, Oteng-Amoako A, Speechley M, Gopaul K, Beauchet O, Annweiler C (2014). The motor signature of mild cognitive impairment: results from the gait and brain study. J Gerontol A Biol Sci Med Sci..

[27] Yang Q, Tian C, Tseng B, Zhang B, Huang S, Jin S (2020). Gait Change in Dual Task as a Behavioral Marker to Detect Mild Cognitive Impairment in Elderly Persons: A Systematic Review and Meta-analysis. Arch Phys Med Rehabil..

[28] Muir SW, Speechley M, Wells J, Borrie M, Gopaul K, Montero-Odasso M (2012). Gait assessment in mild cognitive impairment and Alzheimer’s disease: the effect of dual-task challenges across the cognitive spectrum. Gait Posture..

[29] Lowe DA, MacAulay RK, Szeles DM, Milano NJ, Wagner MT (2019). Dual-Task Gait Assessment in a Clinical Sample: Implications for Improved Detection of Mild Cognitive Impairment. J Gerontol Ser B Psychol Sci Soc Sci..

[30] Lee J, Park S (2018). Effects of a priority-based dual task on gait velocity and variability in older adults with mild cognitive impairment. J Exerc Rehabil..

[31] Bayot M, Dujardin K, Tard C, Defebvre L, Bonnet CT, Allart E (2018). The interaction between cognition and motor control: A theoretical framework for dual-task interference effects on posture, gait initiation, gait and turning. Neurophysiol Clin..

[32] Valkanova V, Ebmeier KP (2017). What can gait tell us about dementia? Review of epidemiological and neuropsychological evidence. Gait Posture..

[33] Nascimbeni A, Caruso S, Salatino A, Carenza M, Rigano M, Raviolo A (2015). Dual task-related gait changes in patients with mild cognitive impairment. Funct Neurol..

[34] Ansai JH, Andrade LP, Rossi PG, Takahashi ACM, Vale FAC, Rebelatto JR (2017). Gait, dual task and history of falls in elderly with preserved cognition, mild cognitive impairment, and mild Alzheimer’s disease. Braz J Phys. Ther..

[35] Buckley C, Alcock L, McArdle R, Rehman R, Del Din S, Mazzà C (2019). The Role of Movement Analysis in Diagnosing and Monitoring Neurodegenerative Conditions: Insights from Gait and Postural Control. Brain Sci..

[36] Gillain S, Warzee E, Lekeu F, Wojtasik V, Maquet D, Croisier JL (2009). The value of instrumental gait analysis in elderly healthy, MCI or Alzheimer’s disease subjects and a comparison with other clinical tests used in single and dual-task conditions. Ann Phys Rehabil Med..

[37] De Cock AM, Fransen E, Perkisas S, Verhoeven V, Beauchet O, Remmen R (2017). Gait characteristics under different walking conditions: Association with the presence of cognitive impairment in community-dwelling older people. PLoS ONE..

[38] Hao W, Zhao W, Kimura T, Ukawa S, Kadoya K, Kondo K (2021). Association of gait with global cognitive function and cognitive domains detected by MoCA-J among community-dwelling older adults: a cross-sectional study. BMC Geriatr..

[39] Godfrey A, Brodie M, van Schooten KS, Nouredanesh M, Stuart S, Robinson L (2019). Inertial wearables as pragmatic tools in dementia. Maturitas..

[40] Mc Ardle R, Del Din S, Galna B, Thomas A, Rochester L (2020). Differentiating dementia disease subtypes with gait analysis: feasibility of wearable sensors?. Gait Posture..

[41] Maquet D, Lekeu F, Warzee E, Gillain S, Wojtasik V, Salmon E (2010). Gait analysis in elderly adult patients with mild cognitive impairment and patients with mild Alzheimer’s disease: simple versus dual task: a preliminary report. Clin Physiol Funct Imaging..

[42] Miyazaki T, Kiyama R, Nakai Y, Kawada M, Takeshita Y, Araki S (2021). Relationships between Gait Regularity and Cognitive Function, including Cognitive Domains and Mild Cognitive Impairment, in Community-Dwelling Older People. Healthcare..

[43] Ijmker T, Lamoth CJC (2012). Gait and cognition: the relationship between gait stability and variability with executive function in persons with and without dementia. Gait Posture..

[44] Pau M, Mulas I, Putzu V, Asoni G, Viale D, Mameli I (2020). Smoothness of Gait in Healthy and Cognitively Impaired Individuals: A Study on Italian Elderly Using Wearable Inertial Sensor. Sensors..

[45] Guimarães V, Sousa I, Correia MV (2021). Orientation-Invariant Spatio-Temporal Gait Analysis Using Foot-Worn Inertial Sensors. Sensors..

[46] Guimarães V, Sousa I, Correia MV (2021). A Deep Learning Approach for Foot Trajectory Estimation in Gait Analysis Using Inertial Sensors. Sensors..

[47] von Elm E, Altman DG, Egger M, Pocock SJ, Gøtzsche PC, Vandenbroucke JP (2007). The Strengthening the Reporting of Observational Studies in Epidemiology (STROBE) statement: guidelines for reporting observational studies. PLoS Med..

[48] Vasunilashorn S, Coppin AK, Patel KV, Lauretani F, Ferrucci L, Bandinelli S (2009). Use of the Short Physical Performance Battery Score to predict loss of ability to walk 400 meters: analysis from the InCHIANTI study. J Gerontol A Biol Sci Med Sci..

[49] Bernabeu-Mora R, Medina-Mirapeix F, Llamazares-Herrán E, García-Guillamón G, Giménez-Giménez LM, Sánchez-Nieto JM (2015). The Short Physical Performance Battery is a discriminative tool for identifying patients with COPD at risk of disability. Int J Chronic Obstructive Pulm Dis..

[50] Guimarães V, Oliveira E, Carvalho A, Cardoso N, Emerich J, Dumoulin C (2021). An Exergame Solution for Personalized Multicomponent Training in Older Adults. Appl Sci..

[51] Thomann AE, Goettel N, Monsch RJ, Berres M, Jahn T, Steiner LA (2018). The Montreal Cognitive Assessment: Normative Data from a German-Speaking Cohort and Comparison with International Normative Samples. J Alzheimers Dis..

[52] Nasreddine ZS, Phillips NA, Bédirian V, Charbonneau S, Whitehead V, Collin I (2005). The Montreal Cognitive Assessment, MoCA: A Brief Screening Tool For Mild Cognitive Impairment. J Am Geriatr Soc..

[53] Shega JW, Sunkara PD, Kotwal A, Kern DW, Henning SL, McClintock MK (2014). Measuring cognition: the Chicago Cognitive Function Measure in the National Social Life, Health and Aging Project, Wave 2. J Gerontol B Psychol Sci Soc Sci..

[54] Thomann AE, Berres M, Goettel N, Steiner LA, Monsch AU. Enhanced diagnostic accuracy for neurocognitive disorders: a revised cut-off approach for the Montreal Cognitive Assessment. Alzheimers Res Ther. 2020;12(39). 10.1186/s13195-020-00603-8. 10.1186/s13195-020-00603-8 PMC7140337 32264975

[55] Bowie CR, Harvey PD (2006). Administration and interpretation of the Trail Making Test. Nat Protoc..

[56] Scarpina F, Tagini S. The Stroop Color and Word Test. Front Psychol. 2017;8. 10.3389/fpsyg.2017.00557. 10.3389/fpsyg.2017.00557 PMC5388755 28446889

[57] Elwood RW (1991). The Wechsler Memory Scale-Revised: Psychometric characteristics and clinical application. Neuropsychol Rev..

[58] Guralnik JM, Simonsick EM, Ferrucci L, Glynn RJ, Berkman LF, Blazer DG (1994). A Short Physical Performance Battery Assessing Lower Extremity Function: Association With Self-Reported Disability and Prediction of Mortality and Nursing Home Admission. J Gerontol..

[59] Guralnik JM, Ferrucci L, Simonsick EM, Salive ME, Wallace RB (1995). Lower-Extremity Function in Persons over the Age of 70 Years as a Predictor of Subsequent Disability. New Engl J Med..

[60] Teruya SL, Dimino C, Silverman KD, Mielenz T (2021). Poor Lower Extremity Functioning Is Associated with Modest Increased Incidence of Probable Dementia. Geriatrics..

[61] Ostir GV, Volpato S, Fried LP, Chaves P, Guralnik JM (2002). Reliability and sensitivity to change assessed for a summary measure of lower body function. J Clin Epidemiol..

[62] Studenski S, Perera S, Wallace D, Chandler JM, Duncan PW, Rooney E (2003). Physical Performance Measures in the Clinical Setting. J Am Geriatr Soc..

[63] Pavasini R, Guralnik J, Brown JC, di Bari M, Cesari M, Landi F (2016). Short Physical Performance Battery and all-cause mortality: systematic review and meta-analysis. BMC Med..

[64] Stookey AD, Katzel LI, Steinbrenner G, Shaughnessy M, Ivey FM (2014). The Short Physical Performance Battery as a Predictor of Functional Capacity after Stroke. J Stroke Cerebrovasc Dis..

[65] Bohannon RW, Crouch R (2019). 1-Minute Sit-to-Stand Test: Systematic Review of Procedures, Performance, and Clinimetric Properties. J Cardiopulm Rehabil Prev..

[66] O’Caoimh R, Timmons S, Molloy DW (2016). Screening for Mild Cognitive Impairment: Comparison of “MCI Specific” Screening Instruments. J Alzheimers Dis..

[67] Perkins NJ, Schisterman EF (2006). The Inconsistency of “Optimal” Cut-points Using Two ROC Based Criteria. Am J Epidemiol..

[68] Hosmer DW, Lemeshow S, Sturdivant RX (2013). Applied logistic regression. 3rd ed. No. 398 in Wiley series in probability and statistics.

[69] Musich S, Wang SS, Ruiz J, Hawkins K, Wicker E (2018). The impact of mobility limitations on health outcomes among older adults. Geriatric Nurss..

[70] Soangra R, Lockhart TE (2017). Dual-Task Does Not Increase Slip and Fall Risk in Healthy Young and Older Adults during Walking. Appl Bionics Biomech..

[71] Li C, Verghese J, Holtzer R (2014). A comparison of two walking while talking paradigms in aging. Gait Posture..

[72] Yogev-Seligmann G, Hausdorff JM, Giladi N (2008). The role of executive function and attention in gait. Mov Disord Off J Mov Disord Soc..

[73] Montero-Odasso M, Muir SW, Speechley M (2012). Dual-task complexity affects gait in people with mild cognitive impairment: the interplay between gait variability, dual tasking, and risk of falls. Arch Phys Med Rehabil..

[74] Hunter SW, Divine A, Frengopoulos C, Montero Odasso M (2018). A framework for secondary cognitive and motor tasks in dual-task gait testing in people with mild cognitive impairment. BMC Geriatr..

[75] MacAulay RK, Wagner MT, Szeles D, Milano NJ (2017). Improving Sensitivity to Detect Mild Cognitive Impairment: Cognitive Load Dual-Task Gait Speed Assessment. J Int Neuropsychol Soc..

[76] Pieruccini-Faria F, Black SE, Masellis M, Smith EE, Almeida QJ, Li KZH (2021). Gait variability across neurodegenerative and cognitive disorders: Results from the Canadian Consortium of Neurodegeneration in Aging (CCNA) and the Gait and Brain Study. Alzheimers Dement..

[77] Choi J, Park J, Lee BI, Shin KJ, Yoo S, Kim H (2019). The Correlation between Cognition Screening Scores and Gait Status from Three-Dimensional Gait Analysis. J Clin Neurol..

[78] Verghese J, Robbins M, Holtzer R, Zimmerman M, Wang C, Xue X (2008). Gait dysfunction in mild cognitive impairment syndromes. J Am Geriatr Soc..

[79] Kang HG, Dingwell JB (2008). Separating the effects of age and walking speed on gait variability. Gait Posture..

[80] Lord S, Galna B, Verghese J, Coleman S, Burn D, Rochester L (2013). Independent domains of gait in older adults and associated motor and nonmotor attributes: validation of a factor analysis approach. J Gerontol A Biol Sci Med Sci..

[81] James EG, Conatser P, Karabulut M, Leveille SG, Hausdorff JM, Travison T (2020). Walking Speed Affects Gait Coordination and Variability Among Older Adults With and Without Mobility Limitations. Arch Phys Med Rehabil..

[82] Parihar R, Mahoney JR, Verghese J. Relationship of gait and cognition in the elderly. Curr Transl Geriatr Exp Gerontol Rep. 2013;2(3). 10.1007/s13670-013-0052-7. 10.1007/s13670-013-0052-7 PMC3859437 24349877

[83] Plummer P, Eskes G. Measuring treatment effects on dual-task performance: a framework for research and clinical practice. Front Hum Neurosci. 2015;9. 10.3389/fnhum.2015.00225. 10.3389/fnhum.2015.00225 PMC4412054 25972801

